# Extremely Non-Auxetic Behavior of a Typical Auxetic Microstructure Due to Its Material Properties

**DOI:** 10.3390/ma14247837

**Published:** 2021-12-17

**Authors:** Mikołaj Bilski, Krzysztof W. Wojciechowski, Tomasz Stręk, Przemysław Kędziora, James N. Grima-Cornish, Mirosław R. Dudek

**Affiliations:** 1 Institute of Applied Mechanics, Poznan University of Technology, Jana Pawła II 24, 60-965 Poznań, Poland; mikolaj.bilski@put.poznan.pl; 2 Institute of Molecular Physics, Polish Academy of Sciences, Smoluchowskiego 17/19, 60-179 Poznań, Poland; kedziora@ifmpan.poznan.pl; 3 Akademia Kaliska im. Prezydenta Stanisława Wojciechowskiego, Nowy Świat 4, 62-800 Kalisz, Poland; 4 Metamaterials Unit, Faculty of Science, University of Malta, MSD 2080 Msida, Malta; james.n.grima-cornish@um.edu.mt; 5 Institute of Physics, University of Zielona Gora, ul. Szafrana 4a, 65-069 Zielona Góra, Poland; m.dudek@if.uz.zgora.pl

**Keywords:** Monte Carlo simulations, extreme Poisson’s ratio, re-entrant geometry, star-shaped geometry, elasticity, hard discs, binary mixtures, mechanical metamaterials

## Abstract

The re-entrant honeycomb microstructure is one of the most famous, typical examples of an auxetic structure. The re-entrant geometries also include other members as, among others, the star re-entrant geometries with various symmetries. In this paper, we focus on one of them, having a 6-fold symmetry axis. The investigated systems consist of binary hard discs (two-dimensional particles with two slightly different sizes, interacting through infinitely repulsive pairwise potential), from which different structures, based on the mentioned geometry, were formed. To study the elastic properties of the systems, computer simulations using the Monte Carlo method in isobaric-isothermal ensemble with varying shape of the periodic box were performed. The results show that all the considered systems are isotropic and *not auxetic*—their Poisson’s ratio is positive in each case. Moreover, Poisson’s ratios of the majority of examined structures tend to +1 with increasing pressure, which is the upper limit for two-dimensional isotropic media, thus they can be recognized as the *ideal non-auxetics* in appropriate thermodynamic conditions. The results obtained contradict the common belief that the unique properties of metamaterials result solely from their microstructure and indicate that the material itself can be crucial.

## 1. Introduction

Auxetic materials are a group of metamaterials characterized by a negative Poisson’s ratio (PR) [1,2,3]. A negative value of this coefficient leads to an unusual behavior of these structures, such as increasing their transverse dimensions (instead of decreasing) during longitudinal stretching [4]. Despite the fact that the theoretical basis indicating the possibility of achieving negative PR values by the media existed much earlier, an intensive increase in the interest of the scientific community in auxetics falls on the 1980s, when the first theoretical models showing such properties were proposed [5,6,7,8,9,10]. One of the most popular two-dimensional (2D) auxetic models is the re-entrant honeycomb structure, introduced by Gibson et al. [5]. The group of re-entrant geometries was later intensively studied and included, among others, such representatives as star re-entrant [11,12,13], double arrowhead [14,15,16], hierarchical star re-entrant [17] and augmented re-entrant honeycomb [18,19,20]. Analitycally and numerically investigated properties of auxetic materials and structures showed unusual properties, from negative compressibility [21,22], increased hardness and values of contact pressure [23,24], enhanced energy and vibration absorption [25,26], to non-intuitive behaviour [27].

In this paper, we focus on star re-entrant geometries with a 6-fold symmetry axis [11]. The structures studied are 2D multi-body periodic systems consisting of binary hard discs (HD) [28], i.e., of circular particles of two slightly different sizes. The diameters of the different HDs are very close to each other, but even such a small difference has a great impact on elastic properties at high densities. Using HDs of one size as a matrix and the other ones as the nanoinclusions, which are thought of as the core of the structure, we have modeled systems that can be described as atomic star re-entrant structures. As is shown in this paper, the star re-entrant geometry, which in the typical case has an auxetic character (i.e., negative PR) [11], in the described (atomic) binary mixtures, interacting through the hard interaction potential, behaves in the opposite way. PR at high densities of most of the structures studied with this geometry tends to the positive limit of 2D isotropic systems, equal to +1 [29,30], and neither of them is auxetic. It is important to emphasize the importance of the described phenomenon. In many works devoted to metamaterials, one can find the suggestion that their unusual, novel properties result only from their (micro)structure. In this work, we show an example that stands in opposition to this statement, since for a typically auxetic microstructure, we have obtained extremely non-auxetic results. Thus, it turns out that not only the microstructure, but also the material itself can be crucial from the point of view of unusual metamaterial properties.

The structure of the article is as follows. In the next sections, we present the models studied (Section 2) and the method (Section 3) used to examine them. In Section 4, we discuss the results obtained and in the last Section 5, we summarize the conducted research.

## 2. Models

All studied structures consist of HDs—circular particles that interact through the interparticle interaction potential $u_{ij}$, which, in the case of two possible particle diameters, takes the form [31]:

(1) $$u_{ij}=\left\{\begin{array}{cc} \infty , & r_{ij}<{\bar{\sigma }}_{ij},\\ 0, & r_{ij}\geq {\bar{\sigma }}_{ij}, \end{array}\right.$$

where ${\bar{\sigma }}_{ij}=\frac{\sigma_{i}+\sigma_{j}}{2}$ is the closest mutual distance at which particles *i* and *j* with diameters $\sigma_{i}$ and $\sigma_{j}$ can be found. The hard interaction potential is an extreme, although nontrivial type of repulsive interaction, used when the attractive interaction between molecules can be considered very small and represents only a certain disturbance in comparison with the dominant repulsive force. The systems in which particles interact through a hard potential play an important role in modeling the condensed matter phases such as liquids [32], liquid crystals [33], plastic crystals [34], as well as periodic and aperiodic crystals [35]. They can also be used to model colloids [36] and granulates[37,38].

The simulations were conducted for a family of atomic star re-entrant structures, formed using the binary HDs (having one of two possible values of the diameter). The diameters of the HDs are equal to (1±δ)σ, respectively, where σ is the unit of length of the simulated systems and δ=0.0005. The selected value of δ corresponds to the one we used in our previous work on binary systems [28], and its detailed influence on the properties of the examined structures remains beyond the scope of this work. Due to the fact that such a small difference of sizes would be impossible to see with the naked eye, for visualization purposes in the figures in this work, we used black dots (for “larger” atoms with diameters (1+δ)σ) and open circles (for “smaller” atoms with diameters (1−δ)σ). Furthermore, each structure is described by three parameters:

- *l*—specifies the length of the side of the core of the structure,
- *t*—specifies the number of rows of the core discs on the side of the core of the structure,
- *s*—specifies the separation of neighbouring cores of the structure.

**Figure 1:**
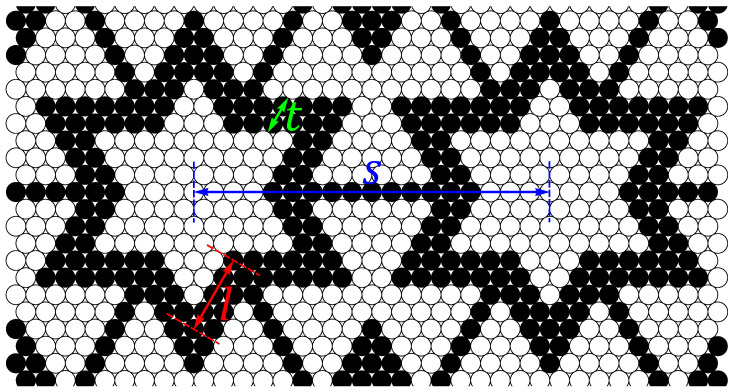
A fragment of the structure S4:2[18]. HDs of different sizes are distinguished by black dots (“larger” atoms with diameters (1+δ)σ) and open circles (“smaller” atoms with diameters (1−δ)σ). The parameters describing the structure are marked with double-sided arrows and letters. Symbol *l* refers to the length of the inner side (i.e., the shortest) of the core that forms the star-like geometry (consisting of black dots in the structure shown). Symbol *t* refers to the number of rows of the core discs on the side of the core. Symbol *s* refers to the separation distance between the central dodecagon atoms (consisting of open circles in the structure shown) surrounded by neighboring cores. It should be noted that *l* and *s* are measured in σ units (assuming δ=0), while *t* is simply the number of “layers” of the side of the core.

The parameters introduced allow for easy identification of the structures in the following part of the work. Each of them will be named using the scheme: “S〈l〉:〈t〉[〈s〉]”. For the convenience of the reader, all of the above parameters are also presented in Figure 1, showing a fragment of the structure S4:2[18].

**Figure 2:**
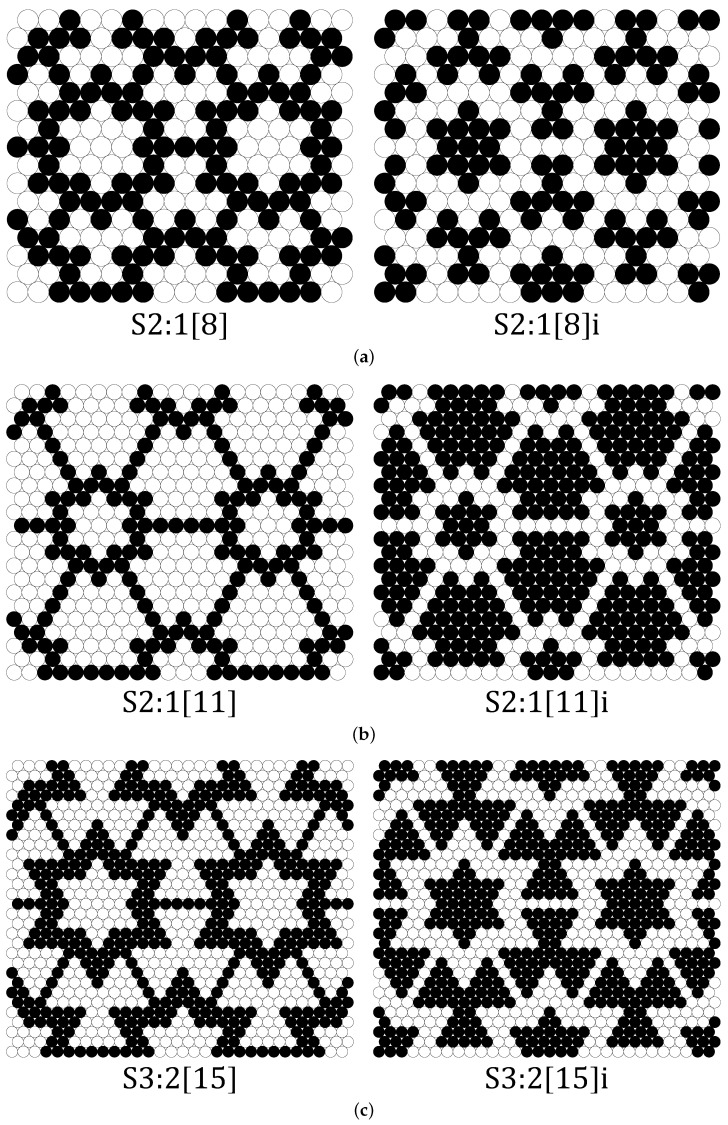
Images of example binary disc systems: (**a**) S2:1[8], (**b**) S2:1[11] and (**c**) S3:2[15]. The “regular” structures are shown in the **left** column, and the corresponding “inverted” structures (with ‘i’ appended to their names) are shown in the **right** column. HDs of different sizes are distinguished by black dots (“larger” atoms with diameters (1+δ)σ) and open circles (“smaller” atoms with diameters (1−δ)σ). The numbers in the names of the structures define their shape and are described in more detail in Figure 1.

The cores of the structure S4:2[18] in Figure 1 are formed by “larger” atoms, illustrated with black dots, while the “smaller” atoms (open circles) constitute the matrix of the structure. In such a case, the structure could be called the “regular” one. In addition to the “regular” structures, we also studied the “inverted” structures, which can be obtained by replacing black dots with open circles (and vice versa) in the “regular” structure. To distinguish the “inverted” structures from the “regular” ones, the letter ‘i’ was appended to their names. Three example structures along with their “inverses” are shown in Figure 2a–c and the rest are included in the Appendix A.

It is worth noting that the presented mechanical models are applicable to novel 2D material systems with similar structural complexity. In particular, the theoretical study conducted in this work can be useful from the point of view of nanostructured, low-dimensional materials with patterned bonding that are intended for mechanical (and, presumably, electronic) applications [39,40].

## 3. Method

There are many methods for the numerical examination of the models developed in this work or their generic analogues. Examples include computational methods such as molecular dynamics [41,42] and the finite element method [43], which have been successfully used by researchers in the field of thermodynamic stability and elasticity. In this work, in order to determine the elastic properties of the structures studied, the Monte Carlo (MC) method in the isobaric-isothermal ensemble (NpT) was used. The method, which was initiated by Parinello and Rahman [44,45,46] is a strain-fluctuation method in which the box of periodicity containing the simulated structure can change its shape. The calculations are based on natural (small) fluctuations of the system (not on artificial deformations), from which one can obtain macroscopic thermodynamic characteristics.

**Figure 3:**
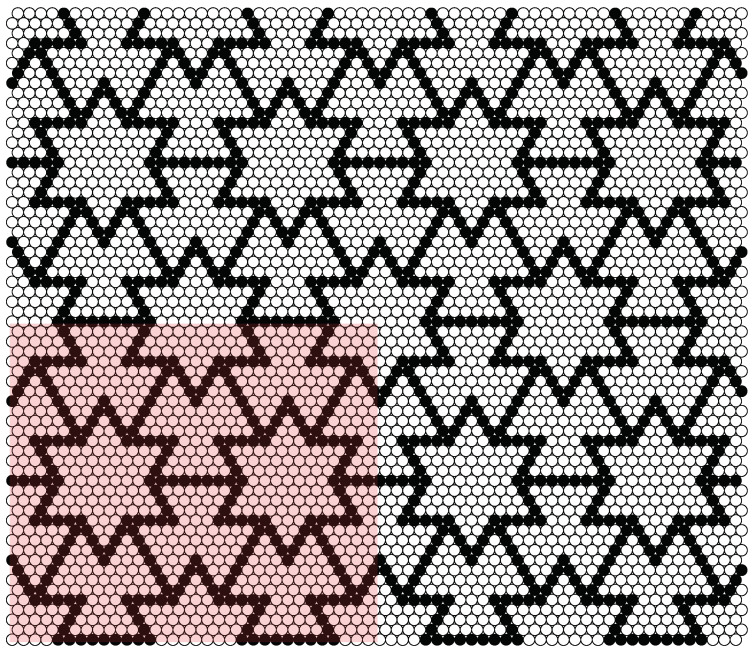
Visualization of the simulation box of S4:1[16] binary disc system and its three periodic images. The pink area indicates the simulation box containing the simulated particles. On the right, above, and in the upper right corner, there are periodic images of the simulation box (similar periodic images could also be drawn in the rest of the area around the simulation box). HDs of different sizes are distinguished by black dots (“larger” atoms with diameters (1+δ)σ) and open circles (“smaller” atoms with diameters (1−δ)σ). The numbers in the name of the structure define its shape and are described in more detail in Figure 1.

The simulation box with its three periodic images is shown in Figure 3, on the example of the S4:1[16] structure. The pink area is the simulation box, and only the particles contained inside it are actually calculated. Due to the periodic boundary conditions, a particle near the boundary of the box can interact with a particle on the opposite side (more precisely, with its periodic image), creating a certain realization of the infinite system.

The shape of the simulation box (Figure 2 and Figure 3) of the studied systems is very close to the rectangle, but in the general case it is a parallelogram, which can be described using a symmetric matrix h, whose columns correspond to the vectors of the box sides [44,45,46]. During the simulation, elements of h can change, which is a practical realization of the fluctuations in shape of the periodic box. Denoting the box matrix of the reference state, H, at a fixed pressure as the average of the box matrix h, H=〈h〉, one can calculate the strain tensor ε at any stage of the simulation (described by h), using equation [46]:

(2) $$\varepsilon =\frac{1}{2}\left(H^{-1}\cdot h\cdot h\cdot H^{-1}-I\right),$$

where I stands for the identity matrix. Averaging the mentioned fluctuations of the shape of the periodic box, expressed by the changes of the strain tensor ε, the elastic compliance tensor S can be calculated:

(3) $$S_{ijkl}=\langle \Delta \varepsilon_{ij}\Delta \varepsilon_{kl}\rangle \frac{V_{p}}{kT},$$

where: *k*—Boltzmann constant, *T*—temperature, $V_{p}=\left|\det\left(H\right)\right|$—average (2D) volume of the system at fixed pressure *p*, and $\langle \cdots \rangle$ is the average in the NpT ensemble. The elastic compliance tensor S contains full information on the elasticity of the system.

The systems studied in this work are isotropic due to their six-fold symmetry [1] and therefore the obtained elements of S should satisfy the conditions:

(4a)Sxxxx=Syyyy=S11,(4b)Sxxyy=Syyxx=S12,(4c)Sxyxy=Sxyyx=Syxxy=Syxyx=S334=S11−S122,(4d)therestofSαβγδnotmentionedabove=0.

In such an isotropic case, the PR is independent of the direction and can be computed in two equivalent ways:

(5a) $$\begin{array}{c} \nu^{\left(1\right)}=-\frac{S_{12}}{S_{11}}\;,\;\mathrm{or} \end{array}$$

(5b) $$\begin{array}{c} \nu^{\left(2\right)}=\frac{S_{33}}{2S_{11}}-1. \end{array}$$

In the case of an anisotropic 2D medium, its PR can be obtained from $S_{\alpha \beta \gamma \delta }$ as a function of a single angle ϕ [47,48]:

(6) $$\nu \left(\phi \right)={\left(1+4\frac{S_{11}+2S_{12}+S_{22}+\left(S_{11}-S_{22}\right)\cos\left(2\phi \right)+\left(S_{13}+S_{23}\right)\sin\left(2\phi \right)}{-S_{11}-6S_{12}-S_{22}+S_{33}+\left(S_{11}-2S_{12}+S_{22}-S_{33}\right)\cos\left(4\phi \right)+2\left(S_{13}-S_{23}\right)\sin\left(4\phi \right)}\right)}^{-1}\;,$$

where, as in Equation (4), Voigt notation was used:

$$\begin{array}{c} S_{11}=S_{xxxx},S_{22}=S_{yyyy},\\ S_{12}=S_{xxyy}=S_{yyxx},\\ S_{13}=2S_{xxxy}=2S_{xxyx}=2S_{xyxx}=2S_{yxxx},\\ S_{23}=2S_{yyyx}=2S_{yyxy}=2S_{yxyy}=2S_{xyyy},\\ S_{33}=4S_{xyxy}=4S_{xyyx}=4S_{yxxy}=4S_{yxyx}. \end{array}$$

The last Equation (6) was used for determination of angular dependence of ν and for independent calculations of the mean PR, $\langle \nu \rangle =\frac{1}{\pi }\int_{0}^{\pi }\nu \left(\phi \right)d\phi$, in order to check for discrepancies with the results obtained with Equation (5).

In the case of an isotropic medium, its elastic properties are fully determined by two independent quantities from the set: bulk modulus, shear modulus, Young’s modulus, and Poisson’s ratio; usually, either bulk modulus and shear modulus or Young’s modulus and Poisson’s ratio are applied. As the work focuses on the matters related to PR, the Young’s modulus, *E*, would be the most appropriate choice for the second quantity. In an isotropic case, it is independent of the direction and, similar to the PR from Equation (5), takes one of two equivalent (dimensionless) forms:

(7a)E*(1)=1S11·σ2kT,or(7b)E*(2)=4S11−S332S11(S11+S12)·σ2kT.

### Simulation Details

In this paper, we examine 16 “regular” structures along with their “inversions” (32 structures in total). The systems can be divided into 5 groups depending on the size of their cores (parameters *l* and *t*, see Figure 1): S2:1[$s_{2:1}$], S3:1[$s_{3:1}$], S4:1[$s_{4:1}$], S3:2[$s_{3:2}$] and S4:2[$s_{4:2}$]. The value of the last parameter, $s_{l:t}$, specifying the separation between neighboring cores, depended on the group and took integer values from the following ranges: $s_{2:1}\in \left[8,11\right]$, $s_{3:1}\in \left[11,14\right]$, $s_{4:1}\in \left[14,17\right]$, $s_{3:2}\in \left[14,15\right]$ and $s_{4:2}\in \left[17,18\right]$. Images of all the studied structures are included in the Appendix A.

The sizes of the simulated systems varied, depending on the parameter *s*. The number of particles, *N*, contained in the simulation boxes of the systems (see Figure 3) can be obtained from the formula: $N=4s^{2}$, therefore, it varied from N=256 in the smallest system to N=1296 in the largest one.

All of the systems were investigated under appropriate thermodynamic conditions, guaranteeing their existence in the crystalline phase. To ensure such conditions, the smallest of the external (reduced) pressure values considered, $p^{*}=\frac{p\sigma^{2}}{kT}$, was (much) higher than the melting point of equidiameter HD system (i.e., with δ=0). Starting with such an assigned value of $p^{*}$, the systems were then increasingly compressed during the simulations, which were conducted for a list of successive pressures, determined by the formula: $p^{*}=10^{1+i/3}$, for integer i∈[1,12].

All structures were simulated in 10 independent runs, each of which had a length of $10^{8}$ cycles (trial steps per particle) for each pressure. The first $10^{7}$ cycles were considered as the equilibrium stage, bringing the simulated systems to the proximity of the equilibrium state at a given pressure; therefore, they were not taken into account by the averaging procedures. The acceptance ratio of the generated trial states was close to 30% for the molecular trial moves and 20% for the box trial moves.

## 4. Results and Discussion

**Figure 4:**
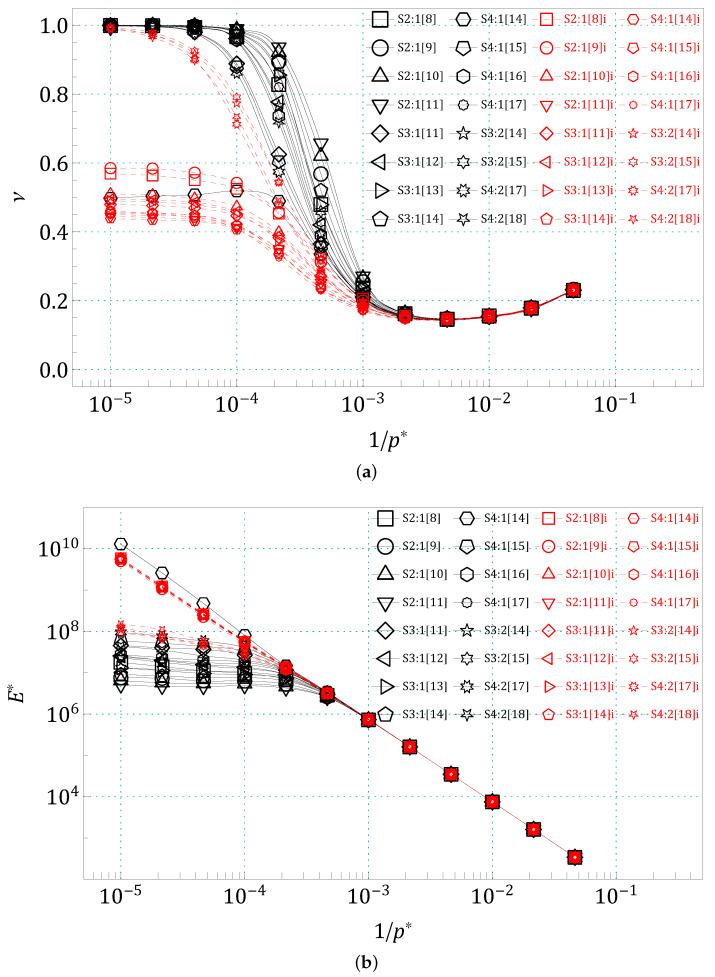
(**a**) The PR values ν and (**b**) the (dimensionless) Young’s modules $E^{*}$ as functions of inverted reduced pressure ${\left(p^{*}\right)}^{-1}$, for all the structures studied. Different colors, black and red, were used to distinguish the “regular” structures (in the **left** column of Figure 2) and their “inverses” (in the **right** column of Figure 2), respectively.

In Figure 4, the results of (a) the PR values and (b) the Young’s modules of the studied systems are shown. As all structures, due to their 6-fold symmetry, are isotropic for small deformations, there are no angular dependencies of their elastic properties. Hence, their PR can be obtained directly from S by Equation (5) (similarly for $E^{*}$, using Equation (7)). Both formulas, Equations (5a) and (5b), gave approximately the same result within an error not exceeding 3%. Simultaneously, it should be noted that all the elements of elastic compliance tensor S were calculated independently, directly from the fluctuations of the h matrix during the simulation, and by no means were the isotropy conditions from Equation (4) artificially enforced. In Appendix A, the angular dependencies of PR are also shown, as well as comparisons of the results obtained with Equation (5) and by averaging Equation (6), where the independence of PR from the direction (isotropy) is clearly visible.

As can be seen in Figure 4a, up to a reduced pressure value of $p^{*}=10^{1+5/3}$, the characteristics of PR as functions of the inverted (dimensionless) pressure, 1/p*, are almost identical for all the systems studied. Above this $p^{*}$, the curves begin to be clearly distinguishable, and many of them converge again to the value ν=+1 at high pressures.

**Figure A1:**
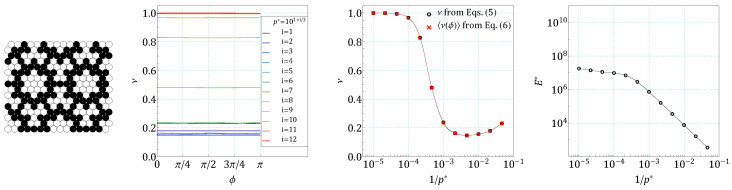
Image of structure S2:1[8] (**leftmost**) and its dependencies: ν(ϕ) (**left**), $\nu (1/p^{*})$ (**center**) and $E^{*}\left(1/p^{*}\right)$ (**right**).

**Figure A5:**
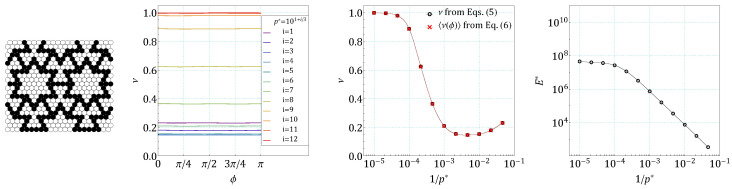
Image of structure S3:1[11] (**leftmost**) and its dependencies: ν(ϕ) (**left**), $\nu (1/p^{*})$ (**center**) and $E^{*}\left(1/p^{*}\right)$ (**right**).

**Figure A9:**
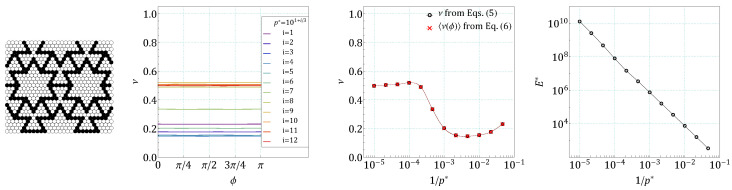
Image of structure S4:1[14] (**leftmost**) and its dependencies: ν(ϕ) (**left**), $\nu (1/p^{*})$ (**center**) and $E^{*}\left(1/p^{*}\right)$ (**right**).

**Figure A13:**
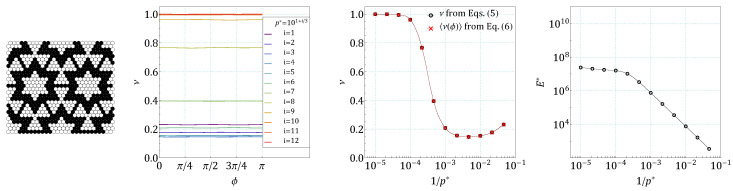
Image of structure S3:2[14] (**leftmost**) and its dependencies: ν(ϕ) (**left**), $\nu (1/p^{*})$ (**center**) and $E^{*}\left(1/p^{*}\right)$ (**right**).

**Figure A15:**
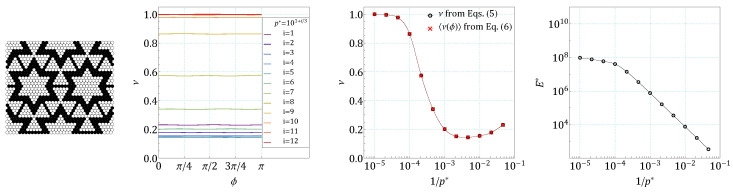
Image of structure S4:2[17] (**leftmost**) and its dependencies: ν(ϕ) (**left**), $\nu (1/p^{*})$ (**center**) and $E^{*}\left(1/p^{*}\right)$ (**right**).

One can see in Figure 4a, that PRs of almost all “regular” structures (black symbols in Figure 4a) tend to the upper limit of 2D isotropic systems, i.e., ν→+1. The only “regular” structure for which PR behaves differently is S4:1[14]—the structure of the S4:1 group, with the smallest separation distance between its cores. The disengagement of this structure from the rest of the “regular” ones seems to be somehow caused by a too small spacing between its cores. As can be seen in Figure A9, the core sites in S4:1[14] are separated only by single *matrix* atoms. Structures with the same core size, but with larger separation between them (even by one single atom, as in S4:1[15]), already tend to ν=+1. It should be noted, however, that similar situations occur in other groups and do not make the PR cease to strive for the upper limit of 2D isotropic systems. The cores of S2:1[8] (Figure A1), S3:1[11] (Figure A5), S3:2[14] (Figure A13) and S4:2[17] (Figure A15) are also separated by single *matrix* atoms, but for $p^{*}\to \infty$, their PR tends to +1.

In the case of the “inverted” structures (red symbols in Figure 4a), i.e., when the core is made of “smaller” atoms and the matrix is made of “larger” ones (see the right column in Figure 2), only the PR of structures with thicker cores (t>1, see Figure 1) tends to +1 with $p^{*}\to \infty$. The PRs of all other examined “inverted” structures tend to some values in the range ν∈(0.435,0.585) at high pressures $p^{*}$.

## 5. Conclusions

In this work, elastic properties of one of the typical auxetic microstructures, the star re-entrant structure, were examined using computer simulations. The simulations were carried out using the Monte Carlo method in the isobaric-isothermal ensemble, which allows one for obtaining complete information on the elastic properties (i.e., all elements of the elastic compliance tensor $S_{\alpha \beta \gamma \delta }$) from the analysis of fluctuations of the shape of the studied systems. The examined systems were binary mixtures consisting of hard discs with two slightly different diameters. The particles of different sizes were used to form the star re-entrant geometry, considering one of them as (nano)inclusions forming the *core* with an assumed shape and the other as the *matrix*.

Within the framework of the study, a total of 32 structures were considered, differing in the size of their cores and their mutual separation within the structure. Furthermore, for each configuration of binary mixtures, two cases including “regular” and “inverted” structures were considered, differing in the type of atoms (“larger” or “smaller”) used to create the star-shaped cores.

The results indicate that all the studied systems are isotropic (in the limit of small deformations, their properties do not depend on the direction), which was predicted even before the start of the simulations, due to the 6-fold symmetry of the geometry under consideration [1,30]. The analysis of Poisson’s ratio has shown that for the majority of the studied systems it tends to +1 with increasing pressure, which is the upper limit for two-dimensional isotropic media, due to the thermodynamic stability conditions [30]. In the case of the “regular” structures, only one behaves differently, and this is predictably due to the insufficient spacing between the star-shaped cores. Things are different in the group of “inversed” structures. In their case, only four structures—those with thicker sides of the cores (two atomic “layers” instead of one) have PR striving for +1 at high pressure.

Based on the results presented, it can be concluded that the binary mixtures considered with sufficiently spaced star-shaped cores consisting of “larger” atoms, at high pressures are *ideal non-auxetics* (ν≈+1). In the case of structures with cores formed by “smaller” atoms, those with thicker core sides show a similar character, while PRs of the remaining ones tend to some values in the range ν∈(0.435,0.585). The key point, however, is that none of the structures shows an auxetic character (ν<0), while each of them is based on the star re-entrant geometry—one of the typical auxetic microstructures. This constitutes an example against the presumption that the unusual properties of metamaterials are only due to their microstructure. The research carried out clearly shows that the material of the structure itself can also be very important.

## Figures and Tables

**Figure A2:**
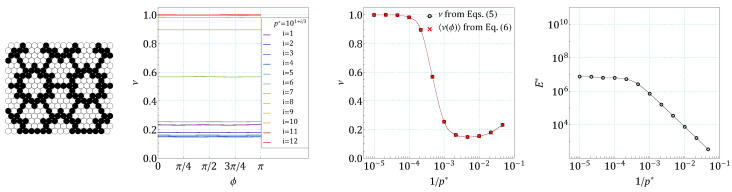
Image of structure S2:1[9] (**leftmost**) and its dependencies: ν(ϕ) (**left**), $\nu (1/p^{*})$ (**center**) and $E^{*}\left(1/p^{*}\right)$ (**right**).

**Figure A3:**
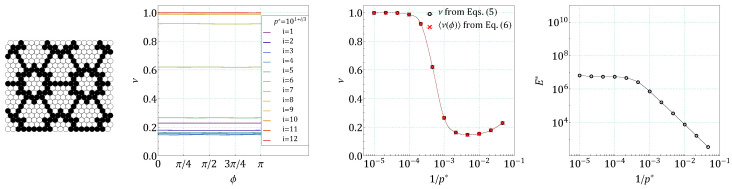
Image of structure S2:1[10] (**leftmost**) and its dependencies: ν(ϕ) (**left**), $\nu (1/p^{*})$ (**center**) and $E^{*}\left(1/p^{*}\right)$ (**right**).

**Figure A4:**
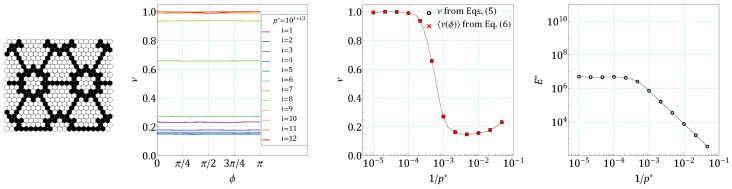
Image of structure S2:1[11] (**leftmost**) and its dependencies: ν(ϕ) (**left**), $\nu (1/p^{*})$ (**center**) and $E^{*}\left(1/p^{*}\right)$ (**right**).

**Figure A6:**
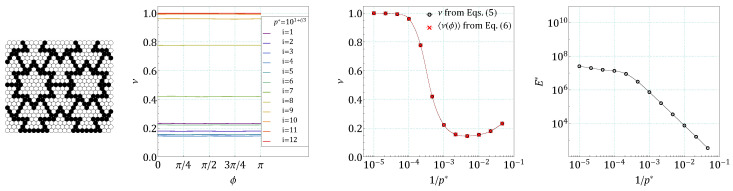
Image of structure S3:1[12] (**leftmost**) and its dependencies: ν(ϕ) (**left**), $\nu (1/p^{*})$ (**center**) and $E^{*}\left(1/p^{*}\right)$ (**right**).

**Figure A7:**
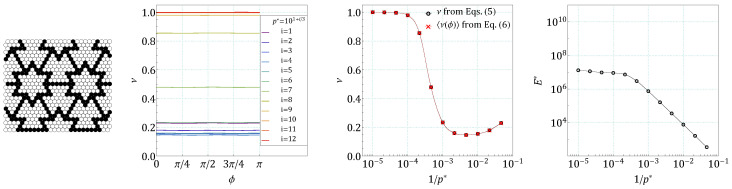
Image of structure S3:1[13] (**leftmost**) and its dependencies: ν(ϕ) (**left**), $\nu (1/p^{*})$ (**center**) and $E^{*}\left(1/p^{*}\right)$ (**right**).

**Figure A8:**
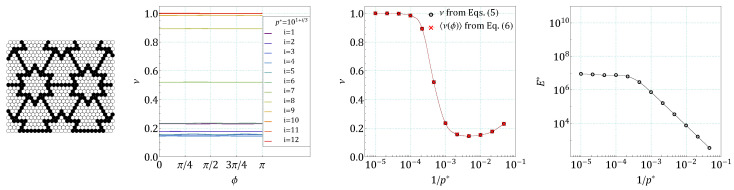
Image of structure S3:1[14] (**leftmost**) and its dependencies: ν(ϕ) (**left**), $\nu (1/p^{*})$ (**center**) and $E^{*}\left(1/p^{*}\right)$ (**right**).

**Figure A10:**
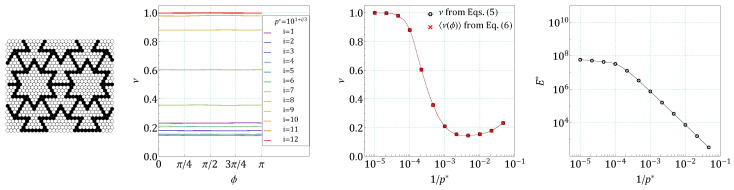
Image of structure S4:1[15] (**leftmost**) and its dependencies: ν(ϕ) (**left**), $\nu (1/p^{*})$ (**center**) and $E^{*}\left(1/p^{*}\right)$ (**right**).

**Figure A11:**
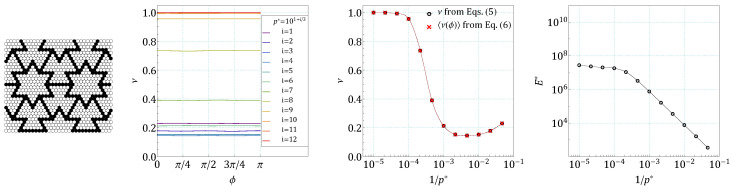
Image of structure S4:1[16] (**leftmost**) and its dependencies: ν(ϕ) (**left**), $\nu (1/p^{*})$ (**center**) and $E^{*}\left(1/p^{*}\right)$ (**right**).

**Figure A12:**
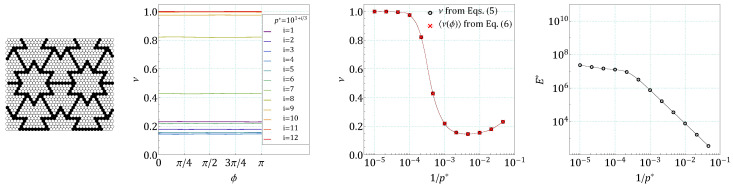
Image of structure S4:1[17] (**leftmost**) and its dependencies: ν(ϕ) (**left**), $\nu (1/p^{*})$ (**center**) and $E^{*}\left(1/p^{*}\right)$ (**right**).

**Figure A14:**
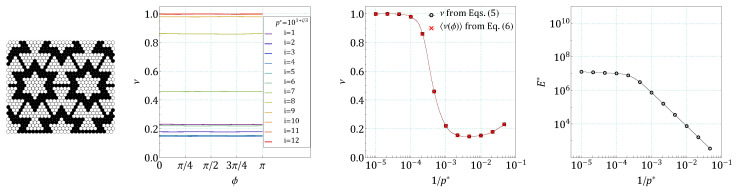
Image of structure S3:2[15] (**leftmost**) and its dependencies: ν(ϕ) (**left**), $\nu (1/p^{*})$ (**center**) and $E^{*}\left(1/p^{*}\right)$ (**right**).

**Figure A16:**
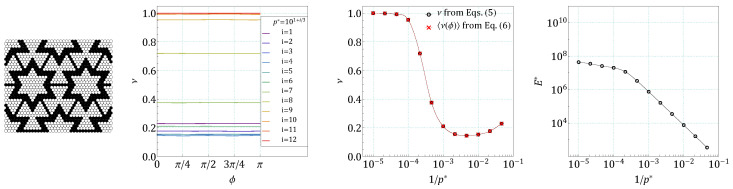
Image of structure S4:2[18] (**leftmost**) and its dependencies: ν(ϕ) (**left**), $\nu (1/p^{*})$ (**center**) and $E^{*}\left(1/p^{*}\right)$ (**right**).

**Figure A17:**
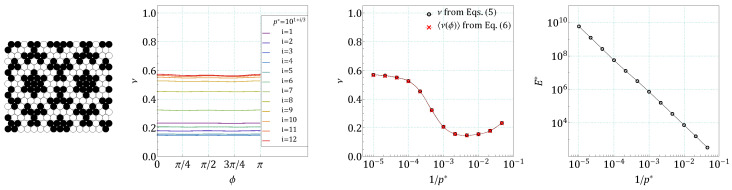
Image of structure S2:1[8]i (**leftmost**) and its dependencies: ν(ϕ) (**left**), $\nu (1/p^{*})$ (**center**) and $E^{*}\left(1/p^{*}\right)$ (**right**).

**Figure A18:**
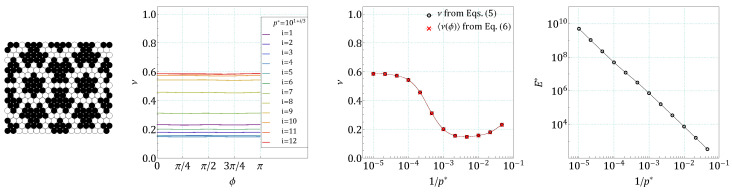
Image of structure S2:1[9]i (**leftmost**) and its dependencies: ν(ϕ) (**left**), $\nu (1/p^{*})$ (**center**) and $E^{*}\left(1/p^{*}\right)$ (**right**).

**Figure A19:**
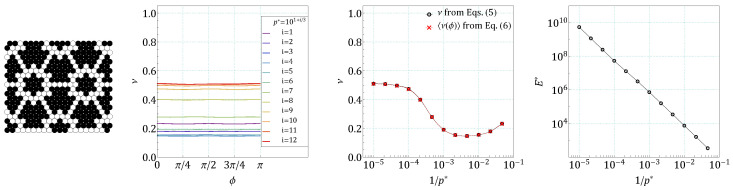
Image of structure S2:1[10]i (**leftmost**) and its dependencies: ν(ϕ) (**left**), $\nu (1/p^{*})$ (**center**) and $E^{*}\left(1/p^{*}\right)$ (**right**).

**Figure A20:**
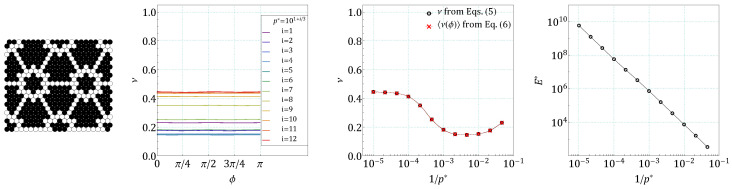
Image of structure S2:1[11]i (**leftmost**) and its dependencies: ν(ϕ) (**left**), $\nu (1/p^{*})$ (**center**) and $E^{*}\left(1/p^{*}\right)$ (**right**).

**Figure A21:**
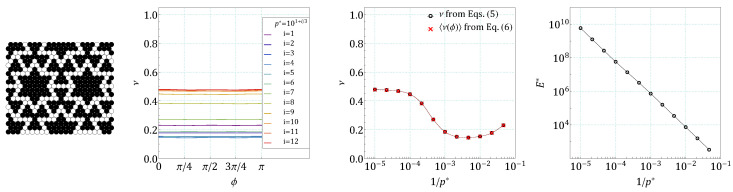
Image of structure S3:1[11]i (**leftmost**) and its dependencies: ν(ϕ) (**left**), $\nu (1/p^{*})$ (**center**) and $E^{*}\left(1/p^{*}\right)$ (**right**).

**Figure A22:**
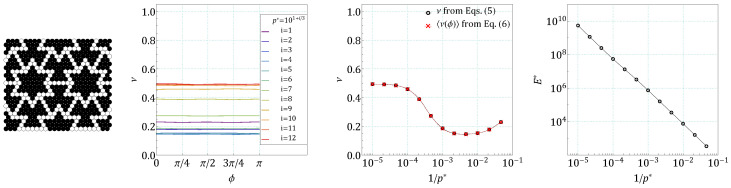
Image of structure S3:1[12]i (**leftmost**) and its dependencies: ν(ϕ) (**left**), $\nu (1/p^{*})$ (**center**) and $E^{*}\left(1/p^{*}\right)$ (**right**).

**Figure A23:**
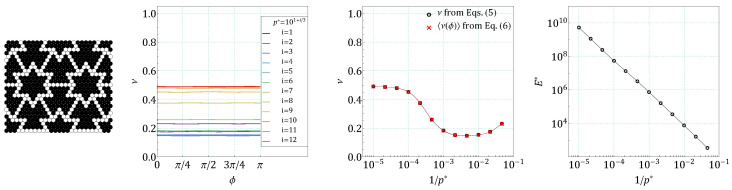
Image of structure S3:1[13]i (**leftmost**) and its dependencies: ν(ϕ) (**left**), $\nu (1/p^{*})$ (**center**) and $E^{*}\left(1/p^{*}\right)$ (**right**).

**Figure A24:**
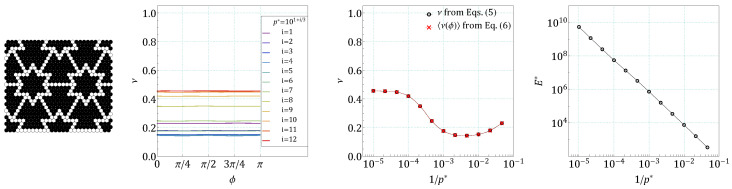
Image of structure S3:1[14]i (**leftmost**) and its dependencies: ν(ϕ) (**left**), $\nu (1/p^{*})$ (**center**) and $E^{*}\left(1/p^{*}\right)$ (**right**).

**Figure A25:**
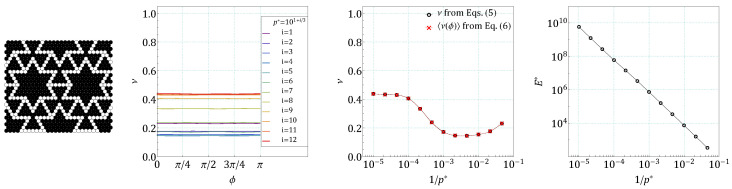
Image of structure S4:1[14]i (**leftmost**) and its dependencies: ν(ϕ) (**left**), $\nu (1/p^{*})$ (**center**) and $E^{*}\left(1/p^{*}\right)$ (**right**).

**Figure A26:**
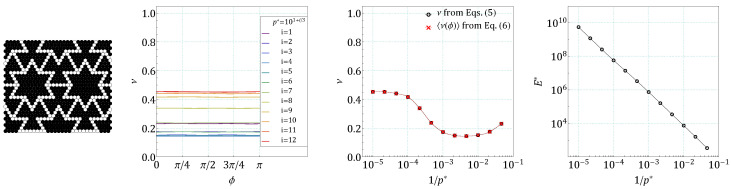
Image of structure S4:1[15]i (**leftmost**) and its dependencies: ν(ϕ) (**left**), $\nu (1/p^{*})$ (**center**) and $E^{*}\left(1/p^{*}\right)$ (**right**).

**Figure A27:**
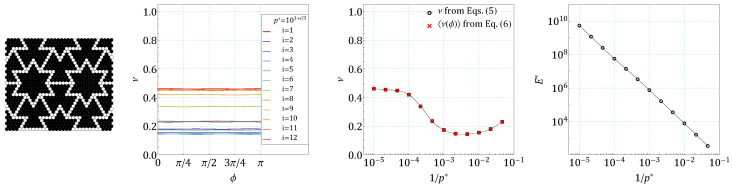
Image of structure S4:1[16]i (**leftmost**) and its dependencies: ν(ϕ) (**left**), $\nu (1/p^{*})$ (**center**) and $E^{*}\left(1/p^{*}\right)$ (**right**).

**Figure A28:**
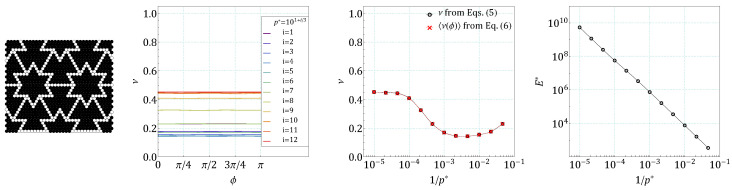
Image of structure S4:1[17]i (**leftmost**) and its dependencies: ν(ϕ) (**left**), $\nu (1/p^{*})$ (**center**) and $E^{*}\left(1/p^{*}\right)$ (**right**).

**Figure A29:**
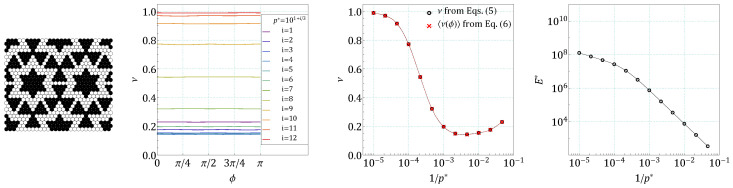
Image of structure S3:2[14]i (**leftmost**) and its dependencies: ν(ϕ) (**left**), $\nu (1/p^{*})$ (**center**) and $E^{*}\left(1/p^{*}\right)$ (**right**).

**Figure A30:**
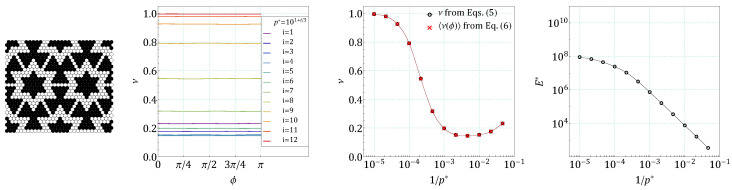
Image of structure S3:2[15]i (**leftmost**) and its dependencies: ν(ϕ) (**left**), $\nu (1/p^{*})$ (**center**) and $E^{*}\left(1/p^{*}\right)$ (**right**).

**Figure A31:**
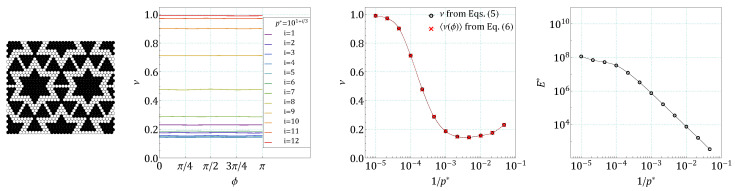
Image of structure S4:2[17]i (**leftmost**) and its dependencies: ν(ϕ) (**left**), $\nu (1/p^{*})$ (**center**) and $E^{*}\left(1/p^{*}\right)$ (**right**).

**Figure A32:**
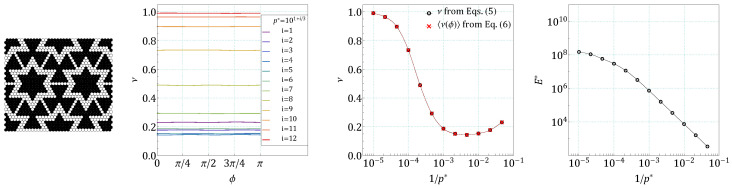
Image of structure S4:2[18]i (**leftmost**) and its dependencies: ν(ϕ) (**left**), $\nu (1/p^{*})$ (**center**) and $E^{*}\left(1/p^{*}\right)$ (**right**).

## Data Availability

The data presented in this study are available on request from the first author (M.B. mikolaj.bilski@put.poznan.pl).

## Abbreviations

The following abbreviations are used in this manuscript:

PR	Poisson’s ratio
2D	two-dimensional
HD	hard disc
MC	Monte Carlo
NpT	isobaric-isothermal ensemble

## Appendix A

In the appendix, the obtained results of angular characteristics of PR and pressure dependences of PR and Young’s modulus, for all the studied structures are shown.
